# Determinants of the quality of life of patients with *NF2*-related schwannomatosis and validation of the Dutch NFTI-QOL questionnaire

**DOI:** 10.1007/s10689-026-00581-0

**Published:** 2026-06-18

**Authors:** Annemijn L. Tops, Dorine Goemans, Emmelien Aten, Josefine E. Schopman, Radboud W. Koot, Hans Gelderblom, Jeroen C. Jansen, Erik F. Hensen

**Affiliations:** 1 Department of Otorhinolaryngology - Head and Neck Surgery, Leiden University Medical Center, 2333 ZA Leiden, The Netherlands; 2 Department of Clinical Genetics, Leiden University Medical Center, 2333 ZA Leiden, The Netherlands; 3 Department of Medical Oncology, Leiden University Medical Center, 2333 ZA Leiden, The Netherlands; 4 Department of Neurosurgery, Leiden University Medical Center, 2333 ZA Leiden, The Netherlands

**Keywords:** *NF2*-related schwannomatosis, Neurofibromatosis, Vestibular schwannoma, Quality of life, Patient-reported outcome measure (PROM)

## Abstract

*NF2*-Related Schwannomatosis (*NF2*-SWN) is a rare tumor predisposition syndrome characterized by heterogeneous clinical manifestations that substantially impact quality of life (QOL). In this diverse patient population, a disease-specific QOL instrument is essential for both research and clinical decision-making. Insight into factors associated with QOL may further facilitate the tailoring of management strategies to individual patient needs. This study aimed to validate the Dutch version of the *NF2* Impact on Quality of Life questionnaire (NFTI-QOL) and to identify factors associated with QOL in Dutch patients with *NF2*. Following forward‑ and backward‑translation, the NFTI-QOL was administered to *NF2*-SWN patients alongside the SF‑36, EQ‑5D and PANQOL. The NFTI-QOL was repeated after one week. Demographic and clinical data were extracted from medical records. To provide clinical context, questionnaires were also administered to patients with sporadic vestibular schwannoma (sVS). 64 *NF2*-SWN and 51 sVS patients completed the questionnaires. The NFTI-QOL demonstrated good validity and reliability. In multivariate linear regression, dizziness (β = 2.533, *p* = 0.027) and facial weakness (β = 4.064, *p* = 0.008) were significantly associated with QOL, whereas hearing complaints were not. Surprisingly, overall QOL did not differ between *NF2*-SWN and sVS patients, whereas patients with sVS even reported poorer hearing-related QOL. The Dutch NFTI-QOL is a reliable instrument for assessing QOL in *NF2-SWN* patients. Dizziness and facial weakness were associated with lower QOL, while hearing problems were not.

**Supplementary Information:**

The online version contains supplementary material available at 10.1007/s10689-026-00581-0.

## Introduction

*NF2*-Related Schwannomatosis (*NF2*-SWN, formerly Neurofibromatosis type 2 or *NF2*) is a rare, autosomal dominant tumor predisposition syndrome with an estimated incidence of 1:50.000 [1]. The hallmark lesions are bilateral vestibular schwannomas (VS) [2]. Additionally, intracranial and spinal meningiomas, ependymomas and schwannomas as well as peripheral schwannomas throughout the body may develop [3]. These different types and locations of tumors result in a variable phenotype. The clinical manifestations of *NF2*-SWN typically include progressive bilateral hearing loss, vestibular dysfunction, and tinnitus, and patients also experience various other neurological deficits affecting vision, mobility, and sensation. This diverse symptomatology leads to substantial variation in disease burden and functional impairment, yet often compromises quality of life (QOL) [4–6].

As curative treatment options are lacking, the aim of *NF2*-SWN management is to prevent or rehabilitate function loss as much as possible, with the ultimate goal of preserving quality of life. Current management options comprise active surveillance, surgery, radiotherapy and pharmacotherapy. Although treatment strategies are mostly guided by the standard objective measures such as tumor progression or the extent of objective neurological function loss, these measures have been shown to have limited correlation with the patients’ QOL [7]. Moreover, the therapy itself may negatively impact QOL. This highlights the need for a comprehensive understanding of the factors that influence QOL in *NF2*-SWN patients and for tools that reliably capture this.

QOL is a multidimensional construct encompassing physical, psychological, social, environmental, functional and personal aspects of well-being [8]. While generic QOL-instruments are aimed at measuring these broader domains, they do not sufficiently reflect the specific multisystem nature of *NF2*-SWN or the problems that have been found most relevant to *NF2*-SWN patients [9]. Disease-specific instruments may therefore provide complementary insight. To address this gap, a *NF2*-SWN-specific questionnaire ‘Neurofibromatosis 2 Impact on Quality of Life’ (NFTI-QOL) was developed in 2012 [10]. This short and concise disease-specific questionnaire has been validated in English and translated into German [11]. As of yet, no validated Dutch version is available. A Dutch version of the NFTI-QOL would support clinical care and research not only in the Netherlands but also in other Dutch speaking *NF2*-SWN populations (for instance in Belgium). Furthermore, QOL in Dutch *NF2*-SWN patients has not yet been systematically investigated. Identifying the factors associated with QOL may guide interventions and supportive care beyond tumor-directed treatment [12].

The aims of this study are to validate the Dutch version of the NFTI-QOL, to deploy it in a Dutch patient cohort and identify factors that determine the quality of life of *NF2*-SWN patients.

## Materials and methods

### Translation of NFTI-QOL into Dutch

The NFTI-QOL was translated into Dutch following the internationally accepted guidelines for translation of patient-reported outcome measures [13, 14]. Two native Dutch speakers with near-native proficiency in English independently performed forward translation. Subsequently, the Dutch version was translated back into English by two native English speakers with near-native proficiency in Dutch. After both translation steps, differences between the two translations were discussed by both translators until consensus was reached. Discrepancies between the backward translation and the original English version were discussed by all translators until resolved. Finally, the resulting Dutch translation was assessed for clarity and readability by an additional native Dutch speaker. The final Dutch version of the NFTI-QOL is provided in the Supplementary Materials (Appendix 1).

### Study design and participants

This prospective study included patients with *NF2*-SWN and a comparison group of patients with a (unilateral) sporadic vestibular schwannoma (sVS). Recruitment took place between July 2025 and February 2026 at the Leiden University Medical Center, a national tertiary referral and expertise center for *NF2*-SWN and VS, and through the Dutch *NF2*-SWN patient association (NFVN).

Inclusion criteria for *NF2*-SWN patients were (1) age ≥ 18 years, (2) a diagnosis of *NF2*-SWN based on the criteria by Plotkin et al. (2022) and (3) a good proficiency in Dutch [2]. All patients known to the *NF2*-SWN expertise center were invited by email, and an open invitation was distributed through the patient association. After providing informed consent, patients received questionnaires either through an online link using Castor EDC [15] or on paper, depending on patient preference.

To provide contextual comparison of QOL domains, a reference group of patients with sVS was recruited. This population was selected because patients experience vestibulocochlear symptoms similar to those in *NF2*-SWN, albeit unilaterally rather than bilaterally, but do not have the additional intracranial and spinal tumors characteristic of *NF2*-SWN. Inclusion criteria for sVS patients were (1) age ≥ 18 years, (2) a diagnosis of unilateral vestibular schwannoma and (3) good proficiency in Dutch. Exclusion criteria were (1) suspected *NF2*-SWN and (2) suffering from visual impairment, neurological disease or neuropathies not related to the vestibular schwannoma. Patients were recruited through the vestibular schwannoma care pathway at the LUMC and approached by email or during a visit at the outpatient clinic. Frequency matching was used to obtain comparable age and sex distributions between both groups.

### Questionnaires

*NF2*-SWN patients received patient-reported outcome measures in addition to a set of questions on demographic and clinical status, including age, age at onset, education level, and hearing rehabilitation. All instruments were sent at once. To assess test–retest reliability, the NFTI-QOL was sent a second time one week after completion of the baseline questionnaires. sVS patients received the same set of questionnaires once. The online and paper versions were identical in content, and the order of questionnaires was fixed for all participants.

The NFTI-QOL is a disease-specific questionnaire consisting of eight items covering different domains of QOL: dizziness/imbalance, hearing problems, facial weakness, sight problems, mobility, outlook on life, pain, and anxiety/depression. Each item is scored on a four-point scale, yielding a total score between 0 and 24, with higher scores reflecting worse QOL [10].

Concurrent validity was assessed using three additional validated patient-reported outcome measures. The 36-Item Short Form Health Survey (SF-36), and the EQ-5D-5L were included as generic health-related QOL-instruments, and the Penn Acoustic Neuroma Quality-of-Life scale (PANQOL) was used as a disease-specific measure relevant to sVS patients [16–18]. All three instruments have validated Dutch versions [19–21]. In these questionnaires, higher scores indicate better functioning.

### Data collection

Clinical information was collected from the patients’ electronic health records when available, and included audiological, neurological, and genetic data. Audiometric evaluation consisted of both pure tone audiometry and word recognition scores (WRS). Pure tone averages (PTA) were calculated by averaging thresholds at 0.5, 1, 2 and 3 kHz. If no measurement was available at 3 kHz, the average of 2 and 4 kHz was used [22, 23]. Facial nerve function was assessed using the House-Brackmann scale [24]. Genetic data were extracted from the clinical genetics report when available and classified according to the validated *NF2*-SWN genetic severity classification by Halliday [25]. In cases of uncertainty regarding interpretation or classification, a Clinical Geneticist with expertise in *NF2*-SWN was consulted. Information on prior treatments, including surgery, radiotherapy and bevacizumab therapy, was collected from the medical record.

The relationship between NFTI-QOL scores and disease burden was assessed using three different measures of disease severity. Clinician-reported severity was derived from a validated symptom-based scoring system and classified into mild, moderate, and severe disease [26]. Genetic severity was classified into five levels according to the validated genetic severity score by Halliday [25]. Patient-reported disease severity was assessed using a single self-reported item included in the questionnaire, in which participants rated the overall impact of *NF2*-SWN on their quality of life as none or mild, moderate, or severe.

### Power calculation

According to the COSMIN (COnsensus-based Standards for the selection of health Measurement Instruments) guidelines, a sample size between 50 and 99 participants is considered adequate for assessing reliability and validity of a patient-reported outcome measurement instrument [27]. We therefore aimed to recruit at least 50 *NF2*-SWN and 50 sVS patients for the validation of the Dutch version of the NFTI-QOL questionnaire.

### Statistical analyses

Descriptive statistics were calculated for demographical and clinical factors. Group differences were calculated using independent samples t-tests and Mann–Whitney U test for continuous variables and chi-squared tests for categorical variables. For correlations, Spearman’s rank correlation coefficients were used. All analyses were performed on a complete‑case basis.

For each questionnaire, standard scoring algorithms were applied to calculate domain, component, and total scores according to the respective scoring manuals [10, 18, 28, 29]. Internal consistency of the NFTI-QOL was assessed using Cronbach’s alpha (α) for the total score. Test–retest reliability was evaluated by calculating the intraclass correlation coefficient (ICC). Concurrent validity was examined by computing Spearman correlation coefficients between total scores of NFTI-QOL and the physical and mental health component summery scores of the SF-36 and the total scores of the EQ-5D and PANQOL. Correlations were interpreted according to the criteria described by Evans [30]. Associations between disease severity and NFTI-QOL scores were evaluated using Spearman’s rank correlation coefficients.

To identify symptoms independently associated with quality of life, a multivariate linear regression model was computed. Because of the limited sample size and to prevent overfitting, a set of predictors was selected a priori based on clinical relevance and existing literature. Assumptions for linear regression were assessed. Additional univariate associations were explored to identify demographic or clinical parameters potentially associated with QOL. As an exploratory contextual comparison, NFTI-QOL domain and total scores were compared between *NF2*-SWN patients and sVS patients.

All statistical analyses were performed with IBM SPSS Statistics for Windows, version 29.0 (released 2022, IBM Corp, Armonk, NY).

### Ethics

The study protocol was approved by the institutional review board and conducted in full compliance with the ethical standard of the 2024 Declaration of Helsinki.

## Results

70 *NF2*-SWN patients and 61 sVS patients provided informed consent. 64 *NF2*-SWN patients (91%) completed the first four questionnaires. 51 patients with sVS completed the questionnaires (84%). There were no missing responses to questions in the surveys.

Baseline characteristics are shown in Table 1. The *NF2*-SWN and sVS groups were comparable in age and sex due to frequency matching. Nine patients (14%) were included from other centers. For these patients, detailed clinical information could not be retrieved as electronic records were not accessible to the researchers. For an additional four patients, records were available but incomplete or outdated. Therefore, descriptive analyses of clinical parameters are based on the remaining 51 patients, unless otherwise specified.

**Table 1:** Demographical and clinical characteristics of the study population

	*NF2*-SWN	VS	*p*-value
Patients, n	64	51	
Female, n (%)^a^	37 (58%)	29 (57%)	0.846^c^
Age in years, mean (SD)^a^	50 (21)	52 (15)	0.446^d^
Age at diagnosis, mean (SD)^a^	36 (21)	49 (16)	< 0.001^d^
Type of prior treatment for *NF2*-SWN related tumors, n (%)^b^			
Active surveillance	64 (100%)	40 (78%)	
Surgery	23 (45%)	8 (16%)	
Radiotherapy	16 (31%)	4 (8%)	
Pharmacotherapy	18 (35%)	0	
Number of prior treatment modalities, n (%)^b^			
No prior treatment	16 (31%)	40 (78%)	
One treatment modality	19 (37%)	10 (20%)	
Two treatment modalities	10 (20%)	1 (2%)	
Three or more treatment modalities	6 (12%)	0	
Hearing rehabilitation, n (%)^a^			
None	28 (44%)	31 (61%)	
Hearing aid	24 (38%)	19 (37%)	
Bone conduction device	1 (2%)	0	
Cochlear implant	2 (3%)	1 (2%)	
Auditory brainstem implant	3 (5%)	0	
Number of patients with specific tumor type, n (%)^b^			
Unilateral VS	9 (18%)	51 (100%)	
Bilateral VS	41 (80%)	0	
Schwannoma (non-VS)	27 (53%)	0	
Meningioma	32 (63%)	0	
Ependymoma	11 (22%)	0	

Abbreviations: n = number; SD = standard deviation; *NF2*-SWN = *NF2*-related schwannomatosis; VS = vestibular schwannoma. ^a^Data available for all 64 *NF2*-SWN participants. ^b^Data available for 51 *NF2*-SWN participants due to missing clinical information. ^c^Calculation was done using chi-squared test. ^d^Calculation was done using independent t-test.

For audiometric analyses, only audiometry performed within two years before the survey were included, as older audiometry was deemed not representative of the current situation due to the frequently progressive nature of hearing loss in this disease. This resulted in 47 *NF2*-SWN patients and 44 sVS patients with evaluable hearing data.

Median total score of the NFTI-QOL in *NF2*-SWN patients was 5.5 (IQR 4–10). NFTI-QOL domain scores for *NF2*-SWN patients are reported in Supplementary Table S1.

Internal consistency was good for the total NFTI-QOL score (α = 0.804). Test–retest reliability was assessed in 61 patients who completed both assessments. The intraclass correlation coefficient (ICC) for the total score was 0.915, indicating excellent reliability. Regarding concurrent validity, total NFTI-QOL scores correlated strongly with total scores of the PANQOL (*ρ* = − 0.755, *p* < 0.001), the physical component summery score (PCS) of the SF-36 (*ρ* = − 0.786, *p* < 0.001) and EQ-5D (*ρ* = − 0.783, *p* < 0.001). There was a significant but moderate correlation to the mental health component summery score (MCS) of the SF-36 (*ρ* = − 0.414, *p* < 0.001). Overall, the Dutch NFTI-QOL demonstrated good reliability and validity, supporting its use as a disease-specific QOL instrument for Dutch speaking patients with *NF2*-SWN.

The distribution of patients across *NF2*-SWN disease severity categories is shown in Table 2. Because the ‘severe’ category of the clinician-reported severity score contained only one patient, the moderate and severe groups were combined prior to correlation analyses to avoid unstable estimates and biased estimates. Clinician-reported disease severity and NFTI-QOL scores were strongly correlated (Spearman’s *ρ* = 0.643, *p* < 0.001). Genetic disease severity was not associated with NFTI-QOL scores (Spearman’s *ρ* = 0.272, *p* = 0.053). Patient-reported severity correlated moderately with NFTI-QOL scores (Spearman’s *ρ* = 0.577, *p* < 0.001).

**Table 2:** Disease severity and its correlations with NFTI-QOL scores

Severity score	n (%)	Spearman’ s* ρ*	*p*-value
Clinician-reported severity score^a^		0.643	< 0.001
Grade 1 (mild)	30 (59%)		
Grade 2 (moderate)	20 (39%)		
Grade 3 (severe)	1 (2%)		
Patient-reported severity score^b^		0.577	< 0.001
Grade 1 (mild)	10 (17%)		
Grade 2 (moderate)	30 (52%)		
Grade 3 (severe)	18 (31%)		
Genetic severity score^a^		0.272	0.053
Group 1A: presumed tissue mosaicism	34 (67%)		
Group 1B: confirmed tissue mosaicism	2 (4%)		
Group 2A: mild *NF2*-SWN	6 (12%)		
Group 2B: moderate *NF2*-SWN	7 (14%)		
Group 3: severe *NF2*-SWN	2 (4%)		

Abbreviations: n = number of patients, *NF2*-SWN = *NF2*-related Schwannomatosis, *ρ* = Spearman’s rank correlation coefficient. ^a^Data available for 51 *NF2*-SWN participants due to missing clinical information. ^b^Data available for all 64 *NF2*-SWN participants.

Based on published literature and clinical relevance, hearing, vestibular and facial symptoms were selected a priori for the analysis of factors that determine QOL in *NF2*-SWN patients. In a multivariate regression model adjusted for age and sex, the presence of vestibular problems (B = 2.533, *p* = 0.027) as well as facial symptoms (B = 4.064, *p* = 0.008) remained independently associated with poorer NFTI-QOL scores (Table 3). The presence of hearing problems were not associated with NFTI-QOL scores. The model explained 28% of the variance in NFTI-QOL scores (adjusted R^2^ = 0.279, F(5, 58) = 5.867, *p* < 0.001).

**Table 3:** Multivariable associations between demographics, symptoms and NFTI-QOL score

Factors	B	95% CI	β	*p*-value
Age	0.015	− 0.030–0.059	0.071	0.514
Sex	0.953	− 1.169–3.076	0.109	0.372
Hearing complaints	0.502	− 1.547–2.551	0.054	0.626
Vestibular symptoms	2.533	0.298–4.768	0.292	0.027
Subjective facial symptoms	4.064	1.104–7.025	0.326	0.008

Multivariable linear regression (n = 64). Model statistics: R^2^ = 0.336, adjusted R^2^ = 0.279, F(5,58) = 5.867, *p* < 0.001. Abbreviations: B = unstandardized coefficient, β = standardized coefficient, CI = confidence interval, n = number of patients in analysis.

Exploratory univariate analyses identified additional associations between NFTI-QOL scores and demographic and clinical variables (Table 4). As these analyses were exploratory and not hypothesis-driven, emphasis is placed on the magnitude of the correlations rather than on formal statistical significance testing. Therefore, only 95% confidence intervals are presented in Table 4, while corresponding *p*-values are provided in Supplementary Table S2 for transparency.

**Table 4:** Exploratory associations between specific clinical parameters and NFTI-QOL scores

	Spearman’s *ρ*	95% CI
Sex	0.347	0.103–0.551
Age	− 0.141	− 0.375–0.110
Age at diagnosis	− 0.290	− 0.506– − 0.040
Time since diagnosis	0.336	0.090–0.544
Education level	− 0.175	− 0.405–0.076
Presence of meningioma	0.394	0.134–0.604
Presence of ependymoma	0.221	− 0.050–0.462
Treatment history		
Surgery	0.352	0.090–0.568
Radiotherapy	**0.473**	0.226–0.663
Pharmacotherapy	0.305	0.040–0.531
Number of treatment modalities	**0.526**	0.289–0.702
Objective facial function (HB)	**0.580**	0.343–0.748
Hearing function in best ear		
PTA	0.143	− 0.152–0.415
WRS	− 0.199	− 0.462–0.096
Presence of hearing implant		
CI	0.066	− 0.183–0.307
ABI	0.261	0.012–0.480

Abbreviations: *ρ* = Spearman’s rank correlation coefficient, 95% CI = 95% confidence interval, HB = House-Brackmann facial nerve grading system, PTA = pure tone average, WRS = word recognition score, CI = cochlear implant, ABI = auditory brainstem implant. Correlation coefficients with an absolute value ≥ 0.4 were considered indicative of at least moderate associations and are therefore shown in bold.

Total NFTI-QOL scores were not significantly different between *NF2*-SWN and sVS patients (Table 5). On the domain level, facial, pain and anxiety domains did not differ as well. However, *NF2*-SWN patients reported significantly higher (less favorable) scores in the vestibular, sight, mobility, and outlook on life domains. Surprisingly, scores on the NFTI-QOL hearing domain were significantly lower (more favorable) in *NF2*-SWN patients compared with sVS patients. To further evaluate this finding, speech discrimination was analyzed using word recognition scores (WRS) of both groups, categorized as non-serviceable (< 50%), serviceable (50–80%), and good (> 80%).

**Table 5:** NFTI-QOL scores of *NF2*-SWN and sVS patients

NFTI-QOL items	*NF2*-SWN mean score (SD)	sVS mean score (SD)	*p*-value^a^
Hearing	1.50 (0.84)	1.78 (0.68)	0.031
Vestibular	1.20 (0.88)	0.89 (0.78)	0.040
Facial	0.31 (0.64)	0.19 (0.58)	0.393
Sight	0.66 (0.95)	0.17 (0.45)	0.023
Mobility	0.52 (0.80)	0.17 (0.38)	0.004
Life	1.45 (0.92)	0.89 (0.92)	0.006
Pain	0.75 (0.87)	0.47 (0.74)	0.073
Anxiety	0.56 (0.79)	0.50 (0.81)	0.895
Total	6.95 (4.37)	5.06 (3.22)	0.059

Abbreviations: *NF2*-SWN = *NF2*-related Schwannomatosis, sVS = sporadic vestibular schwannoma, SD = standard deviation. ^a^Calculation was done using Mann–Whitney U test.

Given the difference in tumor laterality, WRS were evaluated separately for the best- and worst-hearing ear. In the *NF2-*SWN cohort, 83% of patients had good hearing in the best ear, 6% had serviceable hearing, and 11% had non-serviceable hearing in their best hearing ear. In contrast, in the sVS cohort, all patients (100%) demonstrated good hearing in the best ear.

Analysis of the worst-hearing ear showed non-serviceable hearing in 41% of *NF2*-SWN patients and in 27% of sVS patients, serviceable hearing in 13% of *NF2*-SWN patients and 16% of sVS patients, and good hearing in 33% and 57%, respectively. In other words, only 33% of *NF2-*SWN patients had good bilateral hearing versus 57% of sVS patients.

## Discussion

This study represents the validation and deployment of a disease-specific QOL instrument for *NF2*-SWN in a *NF2*-SWN patient cohort in the Netherlands. The Dutch translation of the NFTI-QOL demonstrated to be a reliable questionnaire for use in Dutch patients with *NF2*-SWN. The NFTI-QOL was intentionally designed as a concise and clinically practical disease-specific instrument. As a result, not all individual contributing domains of quality of life are captured in detail. Nonetheless, the NFTI-QOL showed strong correlations with both generic and vestibular schwannoma-specific QOL questionnaires, as well as with clinician-reported and patient-reported disease severity, consistent with previous literature [31, 32]. These findings support its ability to capture disease burden on quality of life.

Compared with previously published *NF2*-SWN cohorts from the United Kingdom and Germany, the Dutch cohort showed lower NFTI-QOL scores, indicating a better overall QOL (mean NFTI-QOL: 7.0 vs. 9.4 and 9.2, respectively) [10, 32]. Several factors may contribute to this difference. The Dutch cohort had a higher mean age at diagnosis (36 years) compared to the average age at diagnosis reported in the literature (20–29 years) [33, 34], and a lower rate of inherited *NF2*-SWN (7% vs. 23–56% in the literature) [35, 36]. These factors in themselves may indicate that the disease phenotype may be milder in this Dutch study population. Unfortunately, direct comparisons of the current cohort with the studies from Germany and the United Kingdom with respect to these factors are hampered by limited data on the clinical and genetic characteristics of these cohorts. Even so, the current Dutch cohort indeed comprised more patients with disease classified as mild (59% vs. 18% and 44%). As NFTI-QOL outcomes correlate well with clinician-reported and patient-reported disease severity, differences in the average QOL between these cohorts may be explained by differences in the case-mix. Additionally, general population-based quality of life indices (i.e. the general quality of life index and the specific health care index) are known to be higher in the Netherlands than in the UK or Germany, which may also affect disease specific quality of life [37].

A striking finding is that the overall QOL did not differ between patients with *NF2*-SWN and sVS, as assessed with NFTI-QOL, EQ-5D and SF-36. This is surprising as *NF2*-SWN patients face a more complex multisystem tumor syndrome causing multiple neurological deficits and a more uncertain prognosis, whereas sVS patients suffer from a sporadic tumor typically affecting audiovestibular function only. The absence of this difference in perceived QOL suggests that it does not solely depend on objective disease severity or the multitude of objective functional impairments, but is probably also substantially influenced by the context of the disease, personality traits and coping abilities. Another possible explanation is the difference in time since diagnosis. Because patients with sVS were age-matched, their average age at diagnosis was higher than that of *NF2-SWN* patients, resulting in a shorter time since diagnosis. This may have provided less opportunity for the sVS patients to develop coping strategies and allow psychological adaptation over time.

Our analyses demonstrate that facial and vestibular symptoms are important determinants of disease-specific quality of life in patients with *NF2*-SWN, whereas hearing impairment was not independently associated with overall quality of life. Vestibular dysfunction is highly prevalent in *NF2*-SWN patients, but has not been studied extensively. Better understanding and management of this important factor in *NF2*-SWN patients could potentially improve their QOL. The strong association between facial symptoms and poorer QOL is consistent with the literature [6, 38]. Surprisingly, hearing loss was not identified as an independent determinant of overall QOL in *NF2*-SWN patients. What is more, *NF2*-SWN patients had better scores on the hearing domain of the NFTI-QOL than sVS patients, despite having worse audiometric outcomes. We hypothesize that in sVS patients, unilateral hearing loss may be experienced as a major functional decline because audiovestibular problems are the predominant clinical feature of the disease, while in *NF2*-SWN patients, hearing loss may represent only one of a plethora of neurological deficits that contribute to the overall disease burden, potentially diminishing its relative impact on perceived quality of life.

Evaluation of other potential factors in *NF2*-SWN-related QOL revealed that the number of treatment modalities and radiotherapy as prior treatment were negatively associated with QOL. These findings should be interpreted with caution given the exploratory nature of these analyses, and it remains uncertain whether the observed associations in *NF2*-SWN reflect the impact of treatment on QOL. An alternative explanation could be that the number and type of treatments are indirect indicators of disease severity. The association between *NF2*-SWN-related QOL and radiotherapy has been observed before [6]. To date, data on treatment-related QOL in *NF2*-SWN remain scarce precluding definite conclusions on the effects of different treatment modalities on patient-reported outcomes in *NF2*-SWN. Nonetheless, these findings stress the necessity to carefully weigh the potential risks of active treatment against the expected benefits.

The main limitation of this study is the relatively limited size of the patient cohort, which is inherent to the rarity of *NF2*-SWN. Even so, the number of patients exceeds the required number as stipulated in the COSMIN guidelines for the validation of patient-reported outcome measurement instruments. Moreover, with an estimated prevalence in the Netherlands of 300–400 patients, the study cohort constitutes approximately 20% of the total Dutch *NF2*-SWN population.

An additional limitation is the cross-sectional design that does not allow for evaluation of longitudinal changes. Causality can therefore not be determined when examining contributing factors of QOL. The NFTI-QOL has now been implemented in routine *NF2*-SWN care in our center, enabling longitudinal assessment of QOL and the evaluation of treatment strategies in the future.

Finally, although all known patients were invited to participate, participation bias cannot be ruled out. However, this potential bias may plausibly act in both directions: on the one hand, patients with more severe disease manifestations may be more motivated to participate in a study such as this, while on the other hand the burden associated with study participation may conversely lead these same patients to decline involvement.

## Conclusion

The Dutch NFTI-QOL is a reliable instrument for assessing disease-specific quality of life in Dutch speaking *NF2*-SWN patients. Facial and vestibular symptoms showed strong correlations, while hearing loss was not significantly associated with quality of life. Notably, overall QOL did not differ between *NF2*-SWN and sporadic vestibular schwannoma patients and patients with a sporadic (unilateral) vestibular schwannoma even reported poorer hearing-related QOL despite better objective hearing levels. This suggests that the relationship between QOL and objective physical impairment is complex and other factors play a major role.

## Supplementary Information

Below is the link to the electronic supplementary material.

Supplementary Material 1

## Data Availability

The data that support the findings of this study are not openly available due to privacy restrictions and are available from the corresponding author upon reasonable request.
