# Supplementary material for: Determinants of the quality of life of patients with NF2-related schwannomatosis and validation of the Dutch NFTI-QOL questionnaire
Source: Fam Cancer. 2026 Jun 18;25(3):66. doi: 10.1007/s10689-026-00581-0 (PMC13279275; doi:10.1007/s10689-026-00581-0)
Supplement: Supplementary file 1 — Supplementary Material 1 [file 10689_2026_581_MOESM1_ESM.docx]

**SUPPLEMENTARY MATERIAL**

***Title:*** *Determinants of the* *quality of life of patients with NF2-Related Schwannomatosis and validation of the Dutch NFTI-QOL questionnaire*

***Authors:*** Annemijn L Tops^1^, MD, Dorine Goemans^2^, Emmelien Aten^3^, MD, PhD, Josefine E Schopman^4^, MD, PhD, Radboud W Koot^5^, MD, PhD, Hans Gelderblom^4^, MD, PhD, Jeroen C Jansen^1^, MD, PhD, Erik F Hensen^1^­, MD, PhD

^1^ Department of Otorhinolaryngology - Head and Neck Surgery, Leiden University Medical Center, 2333 ZA, Leiden, The Netherlands

^2^ Department of Otorhinolaryngology - Head and Neck Surgery, Leiden University Medical Center, 2333 ZA, Leiden, The Netherlands

^3^ Department of Clinical Genetics, Leiden University Medical Center, 2333 ZA, Leiden, The Netherlands

^4^ Department of Medical Oncology, Leiden University Medical Center, 2333 ZA, Leiden, The Netherlands

^5^ Department of Neurosurgery, Leiden University Medical Center, 2333 ZA, Leiden, The Netherlands

***Corresponding author:*** AL Tops, a.l.tops@lumc.nl

**Appendix 1: The Dutch translation of the NFTI-QOL**

*
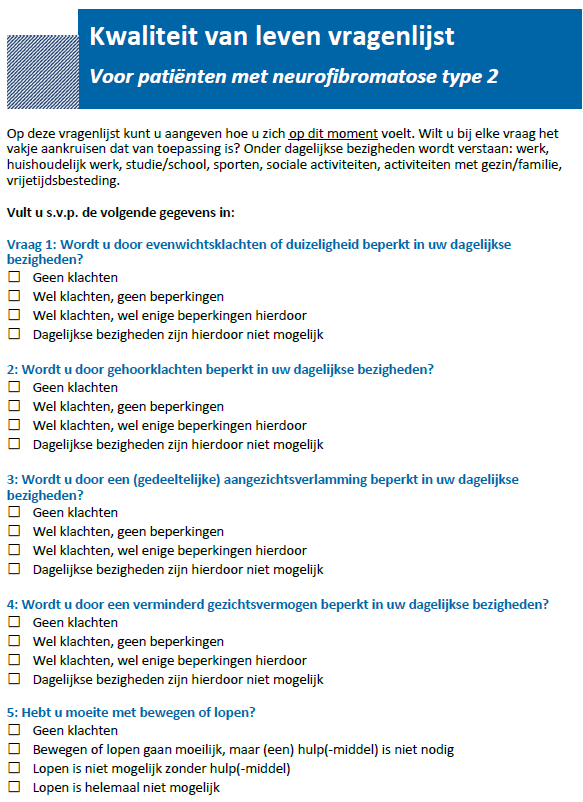

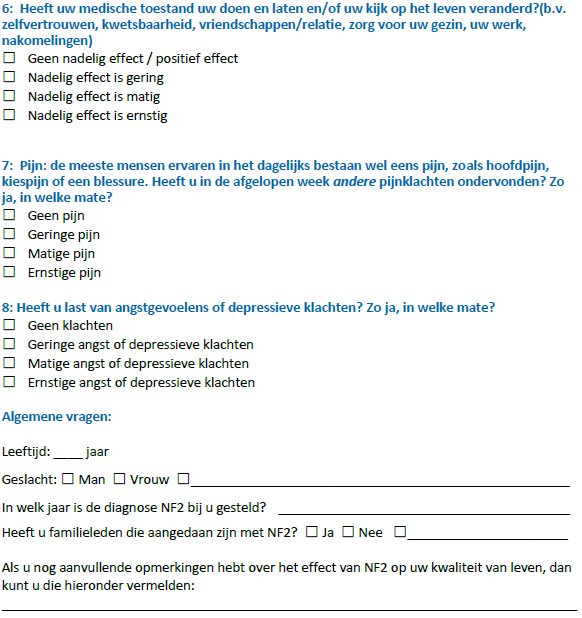
*

**Appendix 2: Table S1. NFTI-QOL domain scores**

| **Table S1. NFTI-QOL domain scores** | | | | |
| --- | --- | --- | --- | --- |
| **Domains** | Not present, n (%) | Yes, but no difficulties, n (%) | Yes, and cause some difficulties, n (%) | Yes, it stops my usual activities, n (%) |
| Q1. Hearing problems | 19 (30) | 13 (20) | 32 (50) | 0 |
| Q2. Dizziness and balance | 11 (17) | 13 (20) | 37 (58) | 3 (5) |
| Q3. Facial weakness | 50 (78) | 8 (13) | 6 (10) | 0 (0) |
| Q4. Sight problems | 40 (63) | 9 (14) | 12 (19) | 3 (5) |
| Q5. Mobility | No problems, n (%) | Some difficulty, can manage on own, n (%) | Unable to walk without help, n (%) | Unable to walk at all, n (%) |
|  | 41 (64) | 15 (23) | 6 (9) | 2 (3) |
| Q6. Outlook on life | Positive or no effect, n (%) | Small negative effect, n (%) | Moderate negative effect, n (%) | Large negative effect, n (%) |
|  | 10 (16) | 24 (38) | 21 (33) | 9 (14) |
| Q7. Pain | 34 (53) | 12 (19) | 18 (28) | 0 (0) |
| Q8. Anxiety and depression | 39 (61) | 15 (23) | 9 (14) | 1 (2) |

**Appendix 3: Table S2. NFTI-QOL domain scores**

| **Table S2. Exploratory associations between specific clinical parameters and NFTI-QOL scores** | | | | | | |
| --- | --- | --- | --- | --- | --- | --- |
|  | | **Spearman’s ρ** | |  | | **95% CI** |
| Sex | | | 0.347 |  | | 0.005 |
| Age | | | -0.141 |  | | 0.270 |
| Age at diagnosis | | | -0.290 |  | | 0.021 |
| Time since diagnosis | | | 0.336 |  | | 0.007 |
| Education level | | | -0.175 |  | | 0.166 |
| Presence of meningioma | | | 0.394 |  | | 0.003 |
| Presence of ependymoma | | | 0.221 |  | | 0.104 |
| Treatment history | | |  |  | |  |
| - Surgery - Radiotherapy - Pharmacotherapy |  | 0.352  **0.473**  0.305 | | | 0.008  <0.001  0.022 | |
| Number of treatment modalities |  | **0.526** | | | <0.001 | |
| Objective facial function (HB) |  | **0.580** | | | <0.001 | |
| Hearing function in best ear   - PTA - WRS |  | 0.143  -0.199 | | | 0.337  0.180 | |
| Presence of hearing implant   - CI - ABI |  | 0.066  0.261 | | | 0.605  0.037 | |
| Abbreviations: ρ = Spearman’s rank correlation coefficient, 95% CI = 95 % confidence interval, HB = House-Brackmann facial nerve grading system, PTA = pure tone average, WRS = word recognition score, CI = cochlear implant, ABI = auditory brainstem implant. Correlation coefficients with an absolute value ≥ 0.4 were considered indicative of at least moderate associations and are therefore shown in bold. | | | | | | |
